# Reframing SARS-CoV-2 evolution in India through an analysis of mutation prevalence across host demographics

**DOI:** 10.1371/journal.pntd.0013191

**Published:** 2025-07-10

**Authors:** Naoko Fujita, Yuki Furuse

**Affiliations:** 1 Department of Microbiology and Infection, The University of Tokyo Pandemic Preparedness, Infection and Advanced Research Center (UTOPIA), The University of Tokyo, Tokyo, Japan; 2 Department of Botany, National Museum of Nature and Science, Tsukuba, Ibaraki, Japan; 3 College of Agriculture, Tamagawa University, Machida, Tokyo, Japan; Zhejiang Wanli University, CHINA

Patel *et al*.’s research offers a valuable contribution to elucidating how SARS-CoV-2 mutational diversity may vary across age groups, geographical regions, and pre- and postvaccination rollout phases in India [1]. The authors conducted an extensive investigation into host-virus interactions at the population level by analyzing 69,975 viral genomes subsampled from 219,149 overall sequences, which were stratified by demographic attributes. Their findings, particularly those regarding the elevated frequency of unique substitutions in working-age adults, prompted important questions about the selective pressures influencing SARS-CoV-2 evolution. In this commentary, we examine these results with methodological rigor, paying particular attention to the authors’ sampling strategies, statistical inferences, and phylogenetic considerations.

Patel *et al*. asserted that there are unique genetic mutations for each group of children, working-age adults, and elderly individuals [1]. Specifically, SARS-CoV-2 genomes from the working-age demographic harbor a greater number of unique substitutions than those in the children and elderly demographics: children, 4,240; working-age adults, 5,429; and elderly, 4,697 (Table 1). This conclusion was derived from a subsampled dataset containing 23,325 randomly selected sequences from each age group to normalize the sample sizes. Although this approach was aimed at maintaining statistical balance, it inadvertently introduced a substantial risk of underrepresenting low-frequency variants. For instance, within the original dataset of working-age adults, which comprised 171,363 sequences, random sampling of 13.6% (23,325/171,363) resulted in an 86.4% probability of omitting a singleton variant. Consequently, variants classified as unique to children or the elderly may have been absent from the working-age adult dataset due to stochastic exclusion during subsampling. This sampling-induced distortion likely led to an overestimation of age-specific substitutions in children and the elderly. Since rare variants may have been excluded purely by chance during random sampling, their absence in certain age groups was not reliable evidence of biological specificity. Therefore, along with the risk of distortion, subsampling also rendered downstream analyses that involved counting the number of viral strains with low-frequency unique mutations statistically unsound.

**Table 1:** Number of unique substitutions across the three age groups.

	Unique substitutions after subsampling by Patel *et al*. [1]	Unique substitutions without subsampling (not considering significance)	Significantly unique substitutions before statistical adjustment	Significantly unique substitutions after Bonferroni correction
Children	4,240	1	1	1
Working-age adults	5,429	2,582	18	0
Elderly	4,697	1	1	1

We performed a reanalysis of the authors’ original dataset without subsampling, using their criteria for unique substitutions (i.e., those found exclusively in one age group). The results revealed a dramatic reduction in such substitutions; there is only one unique substitution in children, 2,582 in working-age adults, and one in the elderly (Table 1). These findings emphasize the necessity of using complete datasets or applying appropriate corrections for sampling probabilities when interpreting frequency-based mutation metrics.

The authors reported that to evaluate statistical significance, they applied a chi-squared test to the distribution of unique substitutions across the age groups with the null hypothesis that all groups had an equal number of unique substitutions. However, this procedure reduced the data to a single-row table, and the resulting test was not a valid chi-squared test. We checked the authors’ computer script (https://github.com/siya-00/SARS-CoV-2_Alchemy/blob/main/script.R) and found that they had actually conducted a Kolmogorov-Smirnov goodness-of-fit test [2]. A proper chi-squared test in this context should assess whether the number of samples carrying each substitution differs significantly across groups by comparing their frequency distributions against variations in group sizes. This approach would remove the need to equalize group sizes through random subsampling, thereby preventing the aforementioned risk of missing low-frequency mutations. For example, the substitution C12138T appeared in 53 sequences in the group of working-age adults but was absent in the groups of children and elderly individuals. The distribution across groups suggests that this was a unique substitution, according to the authors’ definition. A chi-squared test using the actual group sizes (children: 23,325; working-age adults: 171,363; elderly: 24,461) produced a p-value of 0.0025, indicating statistically significant enrichment in the working-age group. Upon applying this method to all unique substitutions detected in our reanalysis, we found that only 1 in children, 18 in working-age adults, and 1 in the elderly exhibited statistical significance at p < 0.05 (Table 1). This means that, when properly tested, most of the unique substitutions reported by Patel *et al*. were not statistically enriched in specific host groups [1].

Equally concerning is the selective application of statistical testing solely to substitutions pre-identified as unique. This approach involved both selection bias and a violation of multiple testing principles. Since the null hypothesis assumes no difference across groups, the authors’ testing of only those seemingly distinct variants inflated the likelihood of a type I error [3,4]. Additionally, the authors performed pairwise comparisons among the three age groups, assuming equal distributions across age groups without adjusting for actual sample population sizes [1]—an issue that parallels the concerns raised earlier.

To investigate this issue, we conducted a comprehensive reanalysis of all substitutions observed in at least five samples from all sequence data, which was in line with the authors’ threshold [1]. We identified 20,000 substitutions overall, regardless of whether they were unique to a specific group. Patel *et al*. based their analysis on the number of unique substitutions observed, without considering the full distribution of all variants [1]. They used a Bonferroni correction with a factor of three to perform a statistical adjustment for multiple testing because they conducted three comparisons for the pairwise test across the three groups. However, since there were 20,000 substitutions to compare, the Bonferroni correction should have had a factor of 20,000 [5].

We performed this adjustment ourselves and found that the number of statistically significant substitutions (adjusted p-value < 0.05) diminished markedly: only one in children, none in working-age adults, and one in the elderly (Table 1). Moreover, the number of sequences containing these significant unique substitutions was very small (children: 6; working-age adults: 0; elderly: 5), rendering them insufficient to support robust demographic inferences. If unique substitutions were defined as mutations whose frequency differed significantly across the three age groups, rather than those uniquely found in one group and absent in the other two (as per Patel *et al*.’s original definition [1]), 520 substitutions would be identified as significant even after the Bonferroni correction.

**Fig 1:**
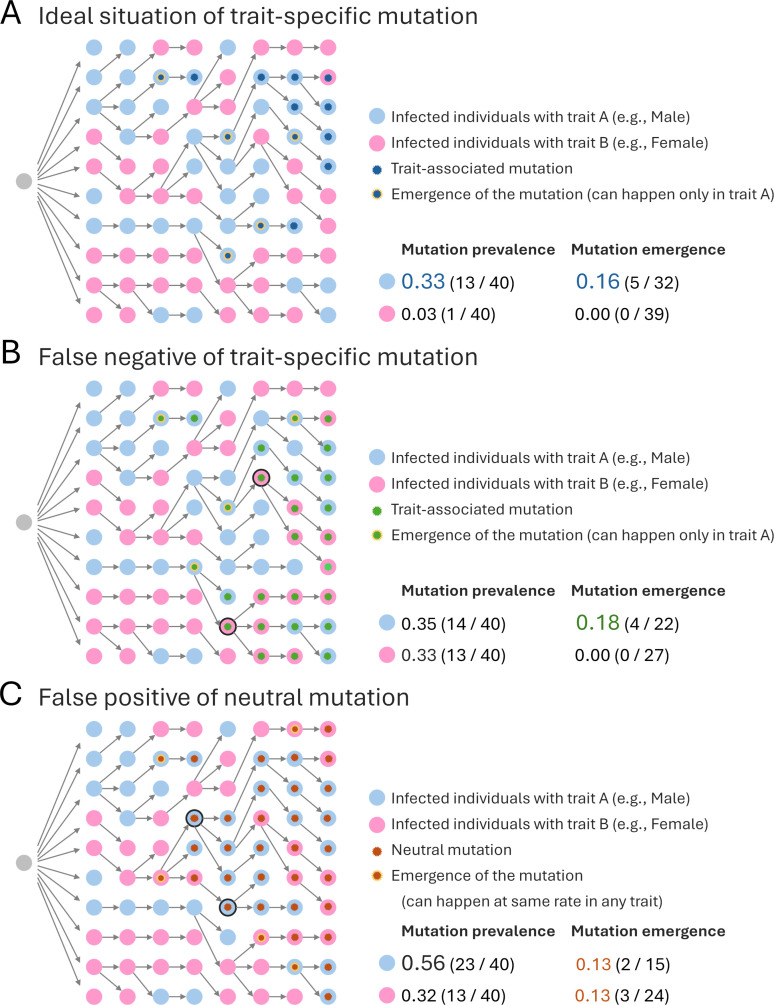
Schematic illustration of trait-associated mutation and its prevalence. The relationship between trait-associated mutation and its prevalence is illustrated. The three panels are explained in detail in the main text.

Another critical limitation of the study arises from the authors’ disregarding the phylogenetic relationships among viral genomes when associating mutations with host characteristics, such as age, sex, or geography [6,7]. The following are some caveats for determining the association of mutations with specific host traits by checking mutation prevalence. Fig 1A illustrates an ideal scenario in which a mutation (blue star) arises exclusively in individuals with a particular host trait (light blue circle); it shows a distinct prevalence of the mutation between groups, allowing for the accurate detection of trait association. Fig 1B presents a false negative case: Although a mutation (green star) originates only in one trait group (light blue circle), it appears in another group (pink circle) at a similar prevalence level due to founder effects initiated by the individuals marked with a black circle; this phenomenon obscures the mutation’s origin and confounds association. Fig 1C demonstrates an example of a false positive: A neutral mutation (brown star) emerges independently of host traits but becomes disproportionately more prevalent in individuals with the light blue trait due to founder effects from the individuals marked with a black circle. These examples collectively illustrate how mutation prevalence can be strongly shaped by transmission dynamics and lineage structures.

**Fig 2:**
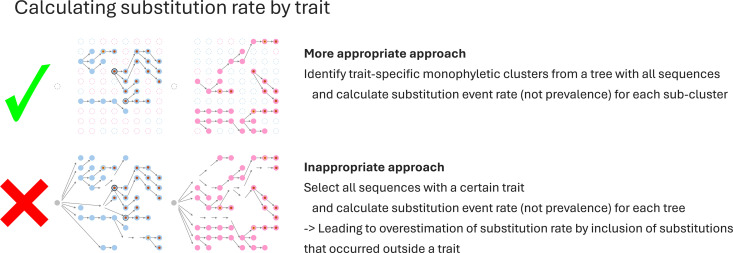
Approaches for checking the association between a mutation and a host trait. Appropriate and inappropriate approaches for checking the association between a mutation and a host trait are illustrated. Detailed explanations of the figure can be found in the main text.

Accurately interpreting host trait–mutation associations requires identifying trait-specific monophyletic clusters within a complete phylogenetic tree and calculating the rate of substitution events (rather than simple prevalence) within each subcluster (Fig 2). When doing so, it should be noted that selecting sequences solely based on the presence of a specific trait and calculating substitution rates for the entire tree with all sequences from that trait can lead to an overestimation of substitution events. Overestimation occurs when substitutions that originate outside the trait-specific lineage are erroneously included, thereby inflating the emergence rate of the substitution in that trait [6,8].

We therefore checked the phylogeny of the Indian SARS-CoV-2 strains with either of the two unique substitutions found in our reanalysis, comparing them with global strains using UShER [9]. The first mutation, A18253G, was detected exclusively in children in the Indian dataset from Patel *et al* [1]. All six viral strains with the mutation belong to a Pango lineage BA.1.617.2 [10]. These six genomes formed a monophyletic cluster when a phylogenetic reconstruction was performed with global sequences. The result indicates that only a single substitution event occurred to generate the mutation in those strains. The second unique mutation, C311G, was found in five genomes from elderly individuals in the original dataset. They are classified as BA.1.1. A phylogenetic analysis suggests that three independent substitution events must have occurred to generate the five strains carrying this mutation. Interestingly, genetically close strains from global samples do not possess the same mutation.

Another area for improvement in Patel *et al*.’s study [1] is the discussion of the functional impacts of identified unique substitutions. Both A18253G and C311G are non-synonymous substitutions resulting in ORF1ab M599V and L16V mutations, respectively. Although no global strains close to the Indian strains have either of the mutations, there are 231 and 57 genomes with the mutations out of ~17 million sequences in the GISAID database as of May 2025. Yet, there are few, if any, published studies regarding those mutations. It appears that Patel *et al*. simply analyzed registered consensus sequences from the database, without considering the methodology used to generate these consensus genomes, such as quality filtering and variant calling [1]. Some unique substitutions may be artifacts introduced during library preparation, sequencing, or data processing.

Patel *et al*.’s study contributes meaningfully to understanding SARS-CoV-2 genomic surveillance and viral evolution [1], but its main conclusions need to be approached with serious caution. The use of random subsampling, flawed statistical methods, and the absence of a phylogenetic analysis undermine the reliability of the associations between SARS-CoV-2 genetic mutations and host demographics. Future work should involve rigorous, unbiased statistics with complete genomic datasets and incorporate phylogenetic structure to better interpret host-virus evolutionary dynamics.

Notably, we were able to identify these methodological limitations and reanalyze the data because of the authors’ decision to openly share the raw data and analysis code. We have also uploaded our analysis to a public repository (https://github.com/yukifuruse1217/india_cov2/blob/main/indiaCOV2_reana_forGit.R). This level of transparency exemplifies the value of open science in fostering critical scrutiny, reproducibility, and the collective advancement of research activities.

The scale of genomic data available for SARS-CoV-2 represents an unprecedented milestone in virology. Never before have researchers had access to such a vast collection of viral genomes, enabling population-scale evolutionary analysis [11]. The tools and expertise needed to fully harness this resource, including virology, epidemiology, statistics, genetics, and evolutionary biology, are still developing. Addressing this challenge will require continued interdisciplinary collaboration.
